# Supplementary material for: Conditional chemoconnectomics (cCCTomics) as a strategy for efficient and conditional targeting of chemical transmission
Source: eLife. 2024 Apr 30;12:RP91927. doi: 10.7554/eLife.91927 (PMC11060718; doi:10.7554/eLife.91927)
Supplement: Figure 3—source data 3. [file elife-91927-fig3-data3.zip › Figure 3 source data3 part 1.zip!/Figure 3-Source data 3-part1/1071f3-1-CRISPResso_Report_jvi77nmx.zip!/Nucleotide_percentage_quilt_around_sgRNA_ACTACTACATCTCCGTGGAG.pdf]

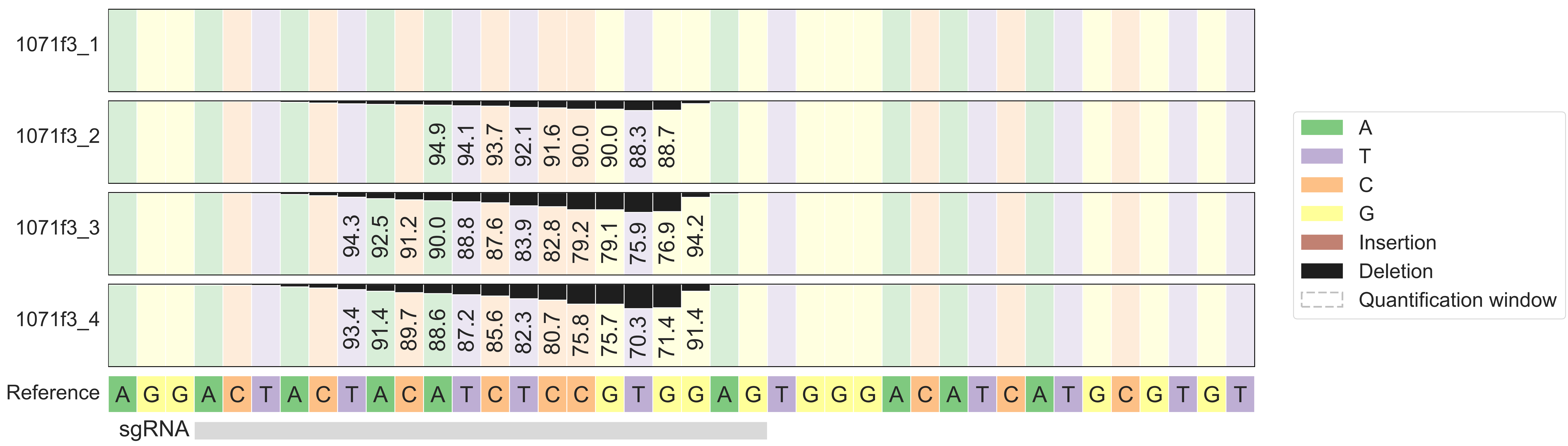

1071f3_1
1071f3_2
1071f3_3
1071f3_4
94.9 94.1 93.7 92.1 91.6 90.0 90.0 88.3 88.7
94.3 92.5 91.2 90.0 88.8 87.6 83.9 82.8 79.2 79.1 75.9 76.9 94.2
93.4 91.4 89.7 88.6 87.2 85.6 82.3 80.7 75.8 75.7 70.3 71.4 91.4
Reference
A G G A C T A C T A C A T C T C C G T G G A G T G G G A C A T C A T G C G T G T
sgRNA
A
T
C
G
Insertion
Deletion
Quantification window
